# Public awareness, knowledge, and attitude regarding proper disposal of unused medicines and associated factors in Gondar city, northwest Ethiopia

**DOI:** 10.3389/fpubh.2024.1372739

**Published:** 2024-06-12

**Authors:** Addisu Afrassa Tegegne, Gebremariam Genet, Liknaw Workie Limenh, Lamrot Yohannes, Abdulwase Mohammed Seid, Tekletsadik Tekleslassie Alemayehu, Wondim Ayenew, Wudneh Simegn

**Affiliations:** ^1^Department of Pharmaceutical Chemistry, School of Pharmacy, College of Medicine and Health Sciences, University of Gondar, Gondar, Ethiopia; ^2^Department of Pharmaceutics, School of Pharmacy, College of Medicine and Health Sciences, University of Gondar, Gondar, Ethiopia; ^3^Department of Environmental and Occupational Health and Safety, Institute of Public Health, University of Gondar, Gondar, Ethiopia; ^4^Department of Clinical Pharmacy, School of Pharmacy, College of Medicine and Health Sciences, University of Gondar, Gondar, Ethiopia; ^5^Department of Social and Administrative Pharmacy, School of Pharmacy, College of Medicine and Health Sciences, University of Gondar, Gondar, Ethiopia

**Keywords:** attitude, knowledge, medication disposal, unused medicines, Ethiopia

## Abstract

**Background:**

Proper disposal of unwanted medicines, in addition to reducing wastage, has a positive impact on the environment and public health. Improper disposal of medications increases the risk of accidental poisonings, particularly among children. This study aimed to assess the level of knowledge, attitudes, and awareness regarding the proper disposal of unused medicines in Gondar city, northwest Ethiopia.

**Method:**

From 30 July to 30 August 2023, a community-based cross-sectional study was conducted among the public in Gondar city. The data were gathered using the Kobo toolbox, exported into an Excel sheet, and then analyzed using SPSS version 27. Multivariate and bivariate binary logistic regressions were performed. A *p*-value of 0.05 with a 95% confidence interval (CI) was used to determine statistical significance.

**Result:**

From 786 study participants, the overall knowledge and attitude of the community toward the proper disposal of unused medicines were 42.6 and 42.9%, respectively. Factors identified in this study included ages between 19 and 25 [AOR = 6.91, 95% CI: (3.45, 13.84); education level: secondary [AOR = 11.82, 95% CI: (1.01, 3.29)] and college and above [AOR = 5.68, 95% CI: (2.25, 14.30)]; prior information [AOR = 6.41; 95% CI: (4.02, 10.22)]; and good attitudes [AOR = 2.11; 95% CI: (1.47, 3.02)]] as factors associated with good knowledge toward proper disposal of unused medicines. In addition, receiving information [AOR = 1.86 95% CI: (1.22, 2.86)], taking medication in the past 6 months [AOR = 1.61, 95% CI: (1.09, 2.38)], and being knowledgeable [AOR = 2.07 95% CI: (1.46, 2.94)] were factors contributing to positive attitudes toward the disposal of unused medicines among the general public. Furthermore, approximately 369 participants (46.9%) in our study lacked awareness about the harmful effects of disposing of unused medicine in regular waste.

**Conclusion:**

A relatively low level of knowledge and attitudes about the proper disposal of unused medications is present in the community. In this regard, a well-coordinated and methodical public awareness campaign is recommended to disseminate information and promote the appropriate disposal of unused medications.

## Introduction

Subsequent advancement in healthcare systems has led to a considerable rise in medication accessibility (1). Medicines are a crucial component of medical care but can cause detrimental effects if wrongly taken and maintained by the consumers. Many consumers are unaware of proper disposal methods for unused medicines (2). Children and pets are at risk of poisoning by unused medicines left insecurely in a home rather than being properly disposed of after use. It is due to the inherent characteristics of pharmaceutical substances that they may inadvertently impact animals and microorganisms within the surrounding ecosystem (3). Drugs may negatively affect wildlife, with estrogens feminizing male fish of many species, raising the risk of ecotoxicological effects (4, 5).

The microorganisms, upon transmission to humans, may already possess resistance to the existing antibiotics due to inadequate disposal, thereby contributing to heightened morbidity and mortality rates, as well as imposing an economic burden on the healthcare system (4, 6). Reducing the risk of releasing unused medicinal products into the environment is highly desirable as the management of pharmaceutical waste in the environment is both challenging and potentially costly, in addition to the harm it poses to public health (7). However, in most African countries, few programs or systems are advocating safe disposal habits of unused medicines (8).

Lack of knowledge about proper disposal systems in low-income settings, coupled with local attitudes, means that pharmaceuticals are often disposed of in an unsafe manner. Programs or systems encouraging the safe disposal of unused medications in African countries remain scarce. For instance, Ethiopia currently lacks a national policy for effectively controlling the safe disposal of unused medicines. However, Ghana has taken steps to implement a program aimed at minimizing the accumulation of unused medications (9). Kenya is considered a benchmark for pharmaceutical waste disposal, with comprehensive guidelines and procedures in place (10). In contrast, Ethiopia faces challenges in pharmaceutical waste management, including a lack of proper practices, limited disposal facilities, inadequate administrative and regulatory systems, and unclear guidelines. Medical waste is often burned at healthcare facilities despite issues with low-quality incinerators and open burning. Furthermore, the safe disposal of pharmaceutical waste is not enforced at the household level in Ethiopia (11). Exploring drug take-back systems and encouraging the participation of consumers in appropriate disposal methods are considered favorable avenues to prevent unused medications from ending up in landfills and wastewater (12). Assessing the extent to which possess the necessary measures to manage pharmaceutical waste effectively is crucial for revamping the wellbeing of individuals and populations (13).

Users’ understanding of pharmaceutical waste and their disposal behavior was assessed in studies conducted around the world. Studies conducted in Africa regarding public awareness of the disposal of unused medicines found that 31.77% (14) and 52.4% (15) “strongly agreed” that unused medicines pose a risk or could lead to negative consequences. While 89.02% of respondents (14) said it was a lack of sufficient information that caused the negative consequences, 38.8% of respondents (15) also believed it was. In Gondar city, studies have revealed that self-medication practices and easy access to prescription medicines have increased significantly in recent years (16, 17). Consequently, medicines may go unused or become outdated and expired and are disposed of indiscriminately. Understanding appropriate medicine disposal and attitudes needs to be developed within communities (18, 19). This study aimed to assess and report the level of knowledge, attitudes, and awareness regarding the proper disposal of unused medicines in Gondar city, northwest Ethiopia.

## Method

### Study settings, design, and period

A community-based cross-sectional study was employed in Gondar city, Northeast Ethiopia. The city is found in Amhara Regional State, 728 km away from Addis Ababa, the capital city of Ethiopia. According to the 2007 population and housing census report, Gondar town has an estimated population of 206,987. The municipal authorities reported a population of 454,445 for the fiscal year 2021/2022. The town is organized into six sub-towns and 36 Kebele administrations. The study took place from 30 July to 30 August 2023.

### Population

The source population for this study comprised all the residents residing in Gondar city. The study focused on residents present during the data collection period. Exclusions were made for individuals under 18 years of age, those facing severe illness, those unable to communicate, and those residing in the city for less than 6 months.

### Sample size determination and sampling technique

**Figure 1:**
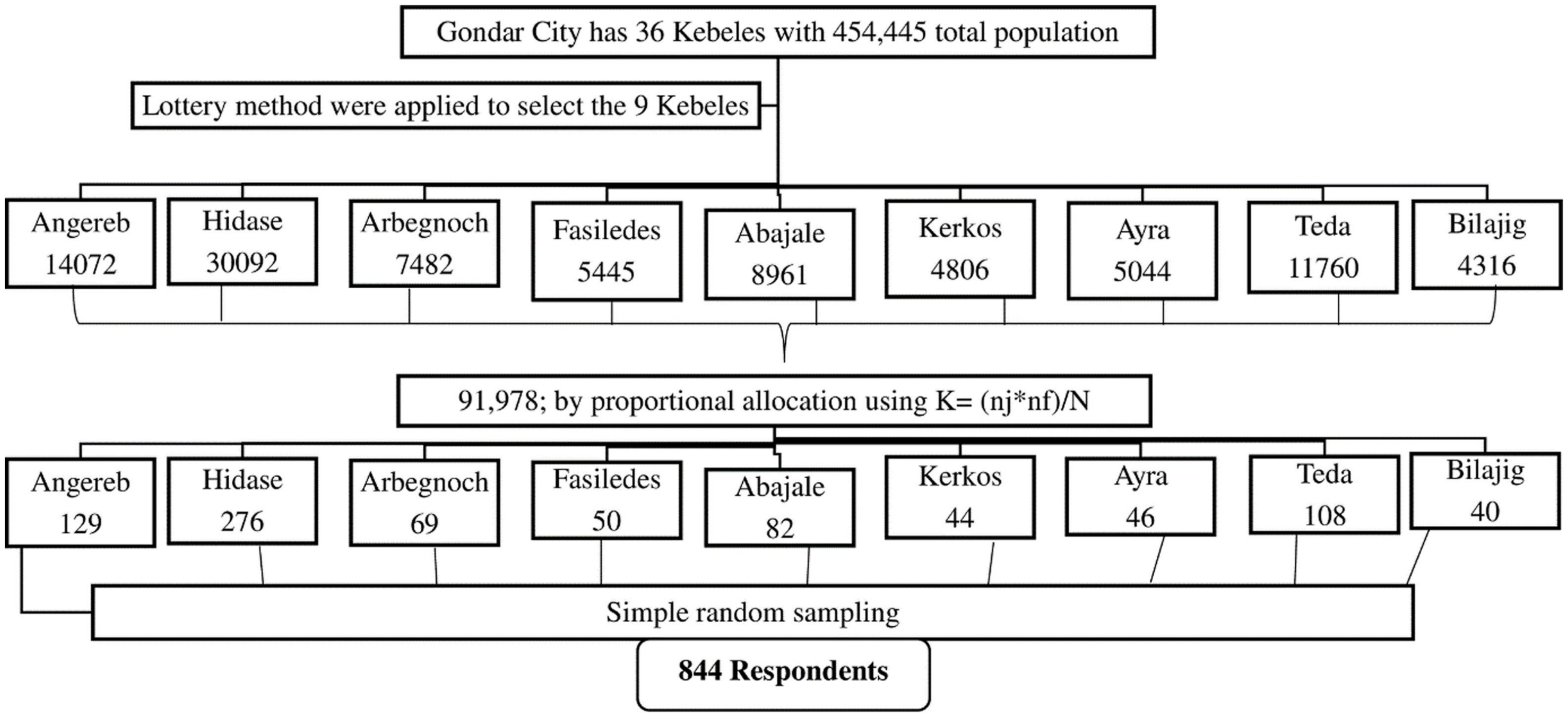
Schematic presentation of the sampling procedure for selecting study participants, Gondar City, northwest Ethiopia, 2023.

The sample size (*n*) of 844 was computed utilizing a single population proportion formula, referencing a prior study in Adigrat city where half of the respondents (50.14%) exhibited good knowledge regarding the disposal of unused and expired medicines (20). This calculation incorporated a 95% confidence interval, a margin of error (d) set at 5%, and an additional 10% for the non-response rate. A design effect was factored in by multiplying the result by 2. The total sample size was proportionately allocated to each kebele through the utilization of a stratified sampling technique. Gondar city encompasses 36 Kebeles distributed among six sub-city administrations. Nine Kebeles (Angereb, Arebegnoch, Fasiledes, Abajale, Kerkos, Ayra, Teda, Bilajig, and Hidase) were randomly selected for inclusion in the study using a lottery method (Figure 1). The present count of the total population was obtained from the city administration. The list of households, along with their corresponding addresses, was acquired from each Kebele administrative office. Within each stratum, samples were selected in proportion to their total population, using the number of households as the sampling frame. A systematic random sampling technique was employed to select households from each Kebele. The sampling interval for each kebele was established by dividing the total number of households in each kebele by its proportionally allocated sample size. Subsequently, every K^th^ value of households was interviewed, with the initial household selected through a lottery method.

### Data collection instrument and data collection procedure

The data collection tool was adapted from prior studies (15, 20–22), and the questionnaire comprised four parts. Part 1 encompassed socio-demographic information, Part 2 included an awareness survey, Part 3 contained items assessing attitudes toward the proper disposal of unused medicines, and Part 4 involved items evaluating knowledge regarding the proper disposal of unused medicines. The questionnaire was initially developed in English and later translated into Amharic, the local language. After adjustments, including the deletion and rephrasing of questions, as well as the addition of instructions, the final questionnaire was compiled. For electronic administration, a computer-based Kobo Toolbox software^1^ was utilized to create the questionnaire, which was structured within Kobo Toolbox for use on mobile devices and tablets during field data collection.

**Table 1:** Socio-demographic characteristics of participants in Gondar city, northwest Ethiopia, 2023 (*n* = 786).

Variables	Categories	Frequency	Percent (%)
Sex	Male	432	55.0
	Female	354	45.0
Age in year*	19–25	166	21.1
	26–30	228	29.0
	31–43	194	24.7
	44+	198	25.2
Marital status	Married	422	53.7
	Single	269	34.2
	Divorced	83	10.6
	Widowed	12	1.5
Educational status	Unable to read and write	103	13.1
	Primary (Grade 1–8)	106	13.5
	Secondary (Grade 9–12)	220	28.0
	College and above	357	45.4
Work	Employee	335	42.6
	Merchant	219	27.9
	Housewife	113	14.4
	Others	119	15.1
Monthly income (ETH-Birr)	up to 4,000	245	31.2
	4,001–6,000	179	22.8
	6,001–8,500	188	23.9
	>8,500	174	22.1

*Mean (±standard deviation); 35.77 (± 13.27) years.

Four individuals with a Bachelor of Science degree in Pharmacy were enlisted for data collection. The data collectors initiated contact with the head of the household for the interview. In instances where the head of the household was unavailable during data collection, any household member aged 18 years and above was selected for participation in the study. If no member of a household was accessible at the time of data collection, the subsequent household in the sampling sequence was chosen. In this study, we used an interviewer-administered questionnaire where the interviewer read the questions and recorded responses using electronic devices, specifically the Kobo Toolbox.

### Variables of the study

#### Dependent variable

Public awareness, knowledge, and attitude toward proper disposal of unused medicines.

#### Independent variables

Socio-demographic factors, such as gender, age, and educational background, prior information, chronic disease history, respondents’ attitudes, and medication usage within the past 6 months.

### Measuring technique

The respondents were invited to answer the following questions about their awareness concerning the proper disposal of unused medicines and respondents’ sense of responsibility toward protecting the environment. (1) Do you have any unused medicine at home? (2) Have you ever received any information about safe ways of disposing of unwanted pharmaceuticals? (3) Where do you get the information? (4) Do you know that the disposing of unused medicine into the waste is harmful? (5) Do you know disposing of unused medicines into waste has any effect on the environment and population health?

A set of 12 yes/no items was used to test respondents’ knowledge of the proper disposal of unused medicines; 1 point was given for the correct answer and 0 points was given for the incorrect answer. The sum of the values was 0 to 12. The mean value was taken as a cutoff point to categorize good and poor knowledge toward proper disposal of unused medicines. A set of 9 Likert-type items running from ‘strongly disagree’ (=1) through ‘neutral’ (=3) to ‘strongly agree’ (=5) concerning respondents’ attitudes was used. The total score ranges from 9 to 45, with an overall higher mean score indicating a positive attitude toward proper disposal of unused medicines.

### Data quality control

The questionnaire was prepared in English and translated into Amharic and then back to English to maintain consistency. To ensure the quality of the data, the questionnaire was checked for its meaningful sentences and incorporation of important points. A pre-test was conducted on 5% of the sample size in non-selected areas of study. We made necessary corrections to address potential issues, unanticipated interpretations, and cultural objections in the questionnaires based on the findings from a pre-test conducted before data collection. The training was given to data collectors on the purpose of the study, the questioner, and how to obtain interviews and fill in the responses.

Furthermore, the principal investigator and supervisor made spot-checking and reviewing electronic-based questionnaires daily to ensure the quality of data completeness and consistency of the information collected. Finally, before data entry, collected data were again reviewed and checked by investigators for completeness, and the data were password-protected on the computer and were only shared with the research team.

### Data analysis

The data were exported into SPSS version 27 for analysis after being cleaned in Excel. Descriptive statistics were calculated to demonstrate means, standard deviations, and frequencies. Responses related to knowledge and attitude were classified into good and poor responses by following the measuring technique mentioned above. The binary logistic regression analysis was carried out to identify variables associated with the dependent variable. The independent variables with a *p*-value less than 0.25 in the bivariable analysis were candidate variables and entered into multivariable logistic regression analysis. Finally, significant factors were identified based on AOR at a 95% confidence level at a *p*-value less than 0.05.

## Results

### Socio-demographic characteristics of respondents

In this study, 786 respondents were interviewed, and the response rate was 93.1%. Of the total respondents, 228 (29.0%) were aged from 26 to 30 years. The mean (± standard deviation) age of mothers was 35.77 (± 13.27) years, and their ages ranged from 19 to 85 years. The majority of the respondents, 335 (42.6%), were employed, and 442 (53.7%) were married. More than half of the respondents, 432 (55.0%), were male. Concerning educational status, almost half of the respondents (357, 45.4%) were college and above, and 103 (13.1%) were unable to read and write (Table 1).

### Status of participants’ awareness and sources of drug disposal-related information

To assess participants’ awareness of the harmful nature of medicines, they were questioned about the effects of improperly disposing of medicines on the environment and population health. The findings indicated that 102 (13%) were unaware of any impact that medicines may have on the environment and population health. In contrast, a significant majority of 684 (87%) demonstrated awareness of the potential consequences of medicines on these aspects (Table 2).

**Table 2:** Drug disposal-related awareness among the study participants in Gondar city, northwest Ethiopia, 2023 (*n* = 786).

Variables	Yes	No
Do you have any unused medicine at home?	275 (35%)	511 (65%)
Do you know disposing of unused medicine into waste is harmful?	417 (53.1%)	369 (46.9%)
Do you know disposing of unused medicines into waste has any effect on the environment and population health?	684 (87%)	102 (13%)

A mere 23.8% of respondents said they had received information about properly disposing of unused medicines. Respondents most often indicated that their sources of information about drug disposal came from the pharmacy (36.9%), mass media (20.9%), and also other sources (24.0%) (Table 3).

**Table 3:** Drug disposal-related information sources among the study participants in Gondar city, northwest Ethiopia, 2023 (*n* = 786).

Variables		Frequency	Percent
Respondents who did not receive any drug disposal-related information		599	76.2
Respondents who had received any drug disposal-related information		187	23.8
Sources of information among these who receive (*n* = 187)	Pharmacy	69	36.9
	Mass-media	39	20.9
	Physicians	24	12.8
	The pharmaceutical industries	10	5.4
	Others	45	24.0

### Knowledge and attitude toward proper disposal of unused medicines

**Figure 2:**
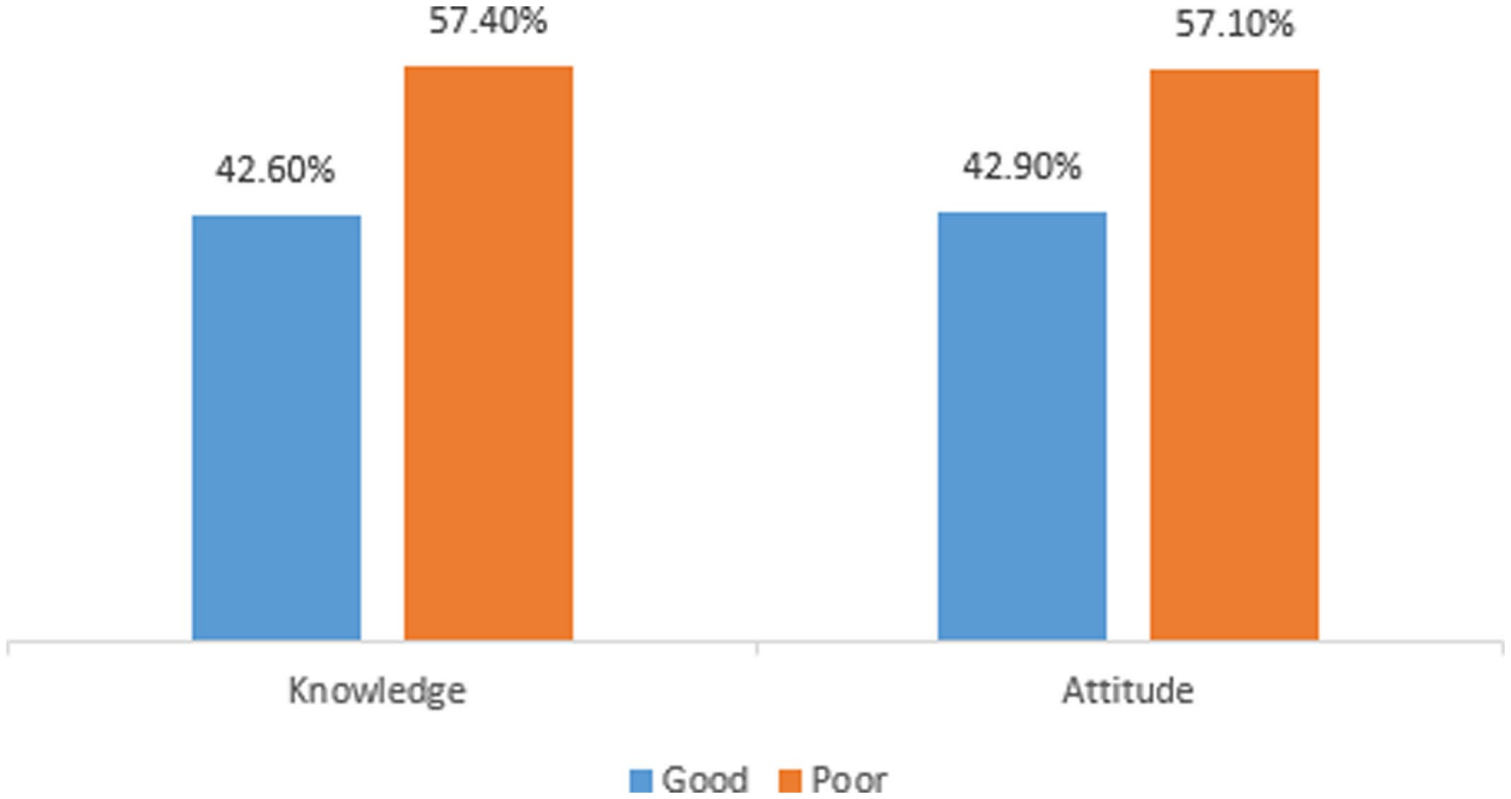
Level of knowledge and attitude toward proper disposal of unused medicines among the public in Gondar City, northwest Ethiopia, 2023 (*n* = 786).

A total of 335 study participants (42.6%; 95% CI: 39.2, 46.3) had good knowledge of how to properly dispose of unused medicines. Approximately 337 (42.9%; 95% CI: 39.4–46.4) participants also exhibited a good attitude toward properly disposing of unused medication (Figure 2).

### Factors associated with respondents’ knowledge of disposal of unused medicines

Age, education status, receiving any information on how to dispose of unwanted pharmaceuticals, medication use in the past 6 months, and attitude toward proper disposal of unused medicines were significantly associated with participants’ knowledge after adjusting for covariates (Table 4). The odds of having good knowledge are 6.91 (AOR = 6.91, 95% CI 3.45, 13.84) and 1.89 (AOR = 1.89, 95% CI 1.15, 3.08) times higher at ages between 19 and 25, and 26 and 30, respectively, as compared to ages older than 44. In comparison to those who were unable to write or read, respondents who were college and above educated and secondary school educated (AOR = 5.68, 95% CI [2.25,14.30]) and (AOR = 11.82, 95% CI [1.01, 3.29]) had good knowledge of proper disposal. It was 6.41 times more likely for participants to have good knowledge if they received information about the safe disposal of unused pharmaceuticals (AOR = 6.41; 95% CI 4.02, 10.22) than their counterparts. In addition, the odds of good knowledge were 2.11 times (AOR = 2.11, 95% CI 1.47, 3.02) higher among those with good attitudes toward proper disposal. In the same vein, possessing good knowledge was found to be 1.83 times higher (AOR = 1.83, 95% CI 1.15, 2.91) among respondents who had not used medication in the past 6 months.

**Table 4:** The bivariable and multivariable logistic regression analysis for factors associated with knowledge of proper disposal of unused medicines among the public in Gondar city, northwest Ethiopia, 2023 (*n* = 786).

Variables	Categories	Knowledge		COR (95% UI)	AOR (95% UI)
		Good (%)	Poor (%)		
Sex	Male	198 (45.8)	234 (54.2)	1.34 (1.01,1.78)	1.08 (0.74,1.57)
	Female	137 (38.7)	217 (61.3)	1	1
Age (years)	19–25	101 (60.8)	65 (39.2)	11.26 (6.64,19.11)	6.91 (3.45,13.84) **
	26–30	131 (57.5)	97 (42.5)	2.26 (1.48,3.45)	1.89 (1.15,3.08) *
	31–43	79 (40.7)	115 (59.3)	1.15 (0.76,1.73)	1.61 (0.99,2.62)
	44+	24 (12.1)	174 (87.9)	1	1
Educational status	Cannot read and write	8 (7.8)	95 (92.2)	1	1
	Primary school (1–8 grade)	26 (24.5)	80 (75.5)	1.59 (1.14,2.23)	1.08 (0.70,1.66)
	Secondary school (9–12 grade)	99 (45.0)	121 (55.0)	4.01 (2.46,6.54)	1.82 (1.01,3.29) ^*^
	College and above	202 (56.6)	155 (43.4)	15.47 (7.30,32.80)	5.68 (2.25,14.30) ^**^
Ever received any information about safe ways of disposing of unwanted pharmaceuticals	No	185 (30.9)	414 (69.1)	1	1
	Yes	150 (80.2)	37 (19.8)	9.07 (6.08,13.52)	6.41 (4.02,10.22) ^**^
Do you have a chronic disease?	Yes	48 (25.1)	143 (74.9)	1	1
	No	287 (48.2)	308 (51.8)	2.78 (1.93,4.00)	0.75 (0.47,1.22)
Do you use any medication for the past 6 months?	No	87 (53.0)	77 (47.0)	1.70 (1.20,2.41)	1.83 (1.15,2.91) *
	Yes	248 (39.9)	374 (60.1)	1	1
Attitude toward proper disposal	Good	192 (57.0)	145 (43.0)	2.83 (3.11,3.80)	2.11 (1.47,3.02) ^**^
	Poor	143 (31.8)	306 (68.2)	1	1

Hosmer and Lemeshow goodness of fit *p* = 0.721, *significant at *p* < 0.05 and **significant at *p* < 0.01.

### Factors associated with respondents’ attitudes toward disposal of unused medicines

Attitude toward proper disposal of unused medicines was significantly associated with ever receiving any information about safe ways of disposing of unwanted pharmaceuticals, use of any medication for the past 6 months, and knowledge about the proper disposal of unused medicines with *p*-values <0.05 and < 0.01. Compared to their counterparts, those who received information about the safe disposal of unused pharmaceuticals were more likely to have a positive attitude toward proper disposal (AOR = 1.86 95% CI: 1.22,2.86). Similarly, respondents who did not use medication in the past 6 months were more likely than respondents who did possess good attitudes (AOR = 1.61, 95% CI 1.09, 2.38). The odds of having a good attitude toward the proper disposal of unused medicines increased (AOR = 2.07 95% CI: 1.46,2.94) when one was knowledgeable about how to dispose of unused medicines (Table 5).

**Table 5:** The bivariable and multivariable logistic regression analysis for factors associated with attitude toward proper disposal of unused medicines among the public in Gondar city, northwest Ethiopia, 2023 (*n* = 786).

Variables	Categories	Attitude		COR (95% UI)	AOR (95% UI)
		Good (%)	Poor (%)		
Sex	Male	205 (47.5)	227 (52.5)	1.52 (1.14,2.02)	1.32 (0.98,1.81)
	Female	132 (37.3)	222 (62.7)	1	1
Educational status	Cannot read and write	47 (45.6)	56 (54.4)	1	1
	Primary school (1–8 grade)	30 (28.3)	76 (71.7)	1.21 (0.78,1.88)	0.54 (0.32,0.90)
	Secondary school (9–12 grade)	80 (36.4)	140 (63.6)	2.58 (1.61,4.12)	1.31 (0.77,2.23)
	College and above	180 (50.4)	177 (49.6)	1.78 (1.26,2.51)	1.23 (0.82,1.83)
Ever received any information about safe ways of disposing of unwanted pharmaceuticals	No	214 (35.7)	385 (64.3)	1	1
	Yes	123 (65.8)	64 (34.8)	3.46 (2.45,4.88)	1.86 (1.22,2.86) ^**^
Do you use any medication for the past 6 months?	No	89 (54.3)	75 (45.7)	1.79 (1.26,2.53)	1.61 (1.09,2.38) *
	Yes	248 (39.9)	374 (60.1)	1	1
Knowledge of proper disposal	Good	192 (57.3)	143 (42.7)	2.83 (3.11,3.80)	2.07 (1.46,2.94) ^**^
	Poor	145 (32.2)	306 (67.8)	1	1

Hosmer and Lemeshow goodness of fit *p* = 0.721, *p < 0.05, and **p < 0.01.

## Discussion

It has been documented in the literature that users are unclear about who offers medication disposal programs and how to properly dispose of unused medications (2). The consumer’s attitude toward medication disposal and their knowledge of the facts about it are essential components of implementation strategies to properly manage pharmaceutical waste (23). Based on this study, only 23.8% of the participants stated that they had received information on the proper disposal of unused medications. This is lower than a similar study, which declared that only 28.3% had never received any information on how to dispose of unused or unwanted medications (24). In the study with participants who received information, pharmacy (36.9%) was most often mentioned as the source of drug disposal information.

A significant finding from our study is that 369 participants (46.9%) showed a lack of awareness regarding the harmful effects of disposing of unused medicine in regular waste. In contrast, countries such as Sweden, where pharmaceutical companies and the government work together to conduct awareness campaigns, have achieved a much higher level of awareness (2). The lack of awareness observed could be partly attributed to the absence of a well-established drug-take-back system in Ethiopia, including the specific study area. Implementing such a program would likely contribute to raising awareness regarding the proper disposal of unused medications (25).

The findings of this study bear witness to another study where 79.8% of respondents thought pharmacists were the best source of information on drug disposal (24). Despite the pharmacy being the best place to educate the community while dispensing (26), the most reported place by respondents, only 42.6% of respondents had good knowledge of how to properly dispose. This might be because pharmacists do not consider proper disposal information a crucial component of counseling, so people are not informed of the proper disposal of unused medications. It may be linked to pharmacists not considering proper disposal information as a vital component of counseling; hence, respondents are not informed by the pharmacist on what to do with unused medications, indicating poor knowledge about proper disposal methods (27).

Based on our data, 42.6% of participants had good knowledge of how to dispose of unused medicines. Compared to a similar study conducted in Adigrat city (20), which gave a score of 50.14, 70.1% in Malaysia (28), 71.6% in Indonesia (29), and 76% in Makerere (30), the value observed in this study was lower. Nevertheless, it was higher in contrast to the findings of research carried out in Lebanon (24), which reported a mere 24.5% level of knowledge. Possibly, the discrepancy in knowledge levels might be attributed to the absence of awareness campaigns promoting proper medicine disposal as beneficial to health and the environment. Furthermore, a lack of convenient and easily accessible locations to return unused medications may also contribute to the lack of knowledge in this area (25).

This study discovered that approximately 42.9% of the participants exhibit a favorable attitude toward the appropriate disposal of unused pharmaceuticals. Conversely, other studies of a similar nature have reported percentages of 82.2% in Adigrat city (20), 76.7% in Malaysia (28), 68.2% in Indonesia (29), 64.8% in India (31), and 22.6% in Lebanon (24). There is probably a need to enhance the attitude of communities concerning the appropriate and secure disposal of unused medications. In addition, it outlines the importance of raising awareness about the detrimental consequences of improper disposal and providing education about the safe disposal of unused medicines.

Age, education, awareness of how to dispose of unused medicines, and medication use in the past 6 months were significantly associated with good knowledge. A good knowledge score was found among respondents of younger ages. Young adults between the ages of 19–25 years are 6.91 times more likely to have good knowledge about the proper disposal of unused medicines than older adults aged 44 years and older, which is in line with a similar study in Lebanon (24). Despite the older participants using more medicines and having more knowledge about the safe disposal of medications, the younger participants scored higher on knowledge. Older adults may be limited in their ability to watch, read, and understand information and lack familiarity with modern technologies, social networks/internet use (32). However, individuals with educational backgrounds at the secondary school level (with a 1.82-fold increase, *p* < 0.05) and at the college level and beyond (with a 5.68-fold increase, *p* < 0.001) exhibited a significantly higher perceived knowledge score compared to those who lack literacy skills. This finding aligns with previous research conducted in Adigrat city (20) and Lebanon (24).

People with higher levels of education may be more intuitive about seeking information and have a better understanding of their surroundings, but those with a lower level of education are more likely to encounter difficulties and may not realize the negative consequences of improperly disposing of medications (18). Participants who received information about the safe disposal of unused medicines were 6.41 times more likely to possess good knowledge than their counterparts. The finding may suggest that the lack of information reaching the general public can have an impact on the optimal approach to unused medicine disposal, influencing individuals’ decisions and promoting the adoption of safe disposal practices (23). Similarly, respondents who had not used medication in the past 6 months were found to have a 1.83 times higher likelihood of possessing good knowledge, which is consistent with a study conducted in Arba Minch (33).

Multivariate logistic regression revealed that good attitudes toward proper disposal of unused medicines were significantly associated with people who did not take any medications in the previous 6 months. Respondents who did not use medication in the past 6 months were 1.61 more likely to possess good attitudes than those who did. Furthermore, individuals who received information about safe disposal of unused pharmaceuticals were 1.86 times more likely to have good attitudes toward proper disposal than their counterparts.

A significant association was found between respondents’ attitudes and their knowledge in the current study. In this study, those with good attitudes were 2.11 times more likely to have good knowledge about the proper disposal of unused medicines, while those with good attitudes were 2.07 times more likely to have a good attitude toward proper disposal of unused medicines, corroborated study conducted in Lebanon (24). This suggests people with positive attitudes are more likely to acquire knowledge, and people with higher levels of knowledge are more likely to have positive attitudes. The idea is that by increasing awareness, the community is more likely to have a positive attitude and knowledge regarding proper disposal, thus helping to protect the environment and public health (34).

### Limitations of the study

A limitation of the study was that the majority of participants were young individuals residing in urban areas, possessing a high level of education and having exposure to information from social media platforms. Therefore, it is necessary to exercise caution when generalizing our findings to the entire population. Although the current sample size may be sufficient for achieving statistical significance, it is advisable to employ a larger sample size to enhance the relevance of the findings to the broader community. Due to the utilization of a cross-sectional study design in this research, establishing a causal association between the dependent and independent variables is not feasible.

## Conclusion

It has been determined that the public possesses a relatively low level of knowledge and attitudes concerning the proper disposal of unused medications. The knowledge and attitudes were associated with multiple factors, which encompass age, educational attainment, reception of information regarding the disposal of unwanted pharmaceuticals, and utilization of any medication within the past half-year. This finding implies that targeted corrective interventions should fully consider these aforementioned factors. Consequently, it is recommended that a well-coordinated and methodical public awareness campaign be executed to disseminate information and knowledge concerning the health hazards associated with unused medications, as well as to promote the appropriate disposal thereof.

## Data availability statement

The data underlying the conclusions in this article will be provided upon request, with inquiries directed to the corresponding author.

## Ethics statement

The study obtained ethical approval from the Department of Pharmacy, College of Medicine and Health Science, Wollo University (Ref: WU Phar/266/2013). Additionally, an official permission letter was obtained from the Gondar city administration to conduct the study. Before obtaining written or verbal consent, potential participants were informed about the study’s objectives and anticipated benefits. All participants willingly took part in the interviews, and their confidentiality was guaranteed.

## Author contributions

AT: Conceptualization, Data curation, Formal analysis, Investigation, Methodology, Project administration, Resources, Software, Supervision, Validation, Visualization, Writing – original draft, Writing – review & editing. GG: Resources, Visualization, Writing – original draft, Writing – review & editing, Data curation, Software. LW: Conceptualization, Data curation, Resources, Visualization, Writing – original draft, Writing – review & editing, Software. LY: Conceptualization, Data curation, Resources, Software, Visualization, Writing – original draft, Writing – review & editing. AM: Data curation, Resources, Software, Visualization, Writing – original draft, Writing – review & editing. TA: Data curation, Resources, Software, Visualization, Writing – original draft, Writing – review & editing. WA: Conceptualization, Data curation, Resources, Software, Visualization, Writing – original draft, Writing – review & editing. WS: Conceptualization, Data curation, Formal analysis, Investigation, Methodology, Project administration, Resources, Software, Supervision, Validation, Visualization, Writing – original draft, Writing – review & editing.

## Footnotes

^1^https://www.kobotoolbox.org/

## References

[1] Ozawa S Shankar R Leopold C Samuel O. Access to medicines through health systems in low-and middle-income countries, vol. 34. Oxford: Oxford University Press (2019). iii1 p. 10.1093/heapol/czz119 PMC6901066 31816069

[2] Paut Kusturica M Tomas A. Disposal of unused drugs: Knowledge and behavior among people around the world. Rev Environ Contam Toxicol. (2017):71–104. doi: 10.1007/398_2016_3 27115675

[3] Kayode-Afolayan SD Ahuekwe EF Nwinyi OC. Impacts of pharmaceutical effluents on aquatic ecosystems. Scientific African. (2022) 17:e01288. doi: 10.1016/j.sciaf.2022.e01288

[4] Desbiolles F Malleret L Tiliacos C Wong-Wah-Chung P Laffont-Schwob I. Occurrence and ecotoxicological assessment of pharmaceuticals: is there a risk for the Mediterranean aquatic environment? Sci Total Environ. (2018) 639:1334–48. doi: 10.1016/j.scitotenv.2018.04.351 29929299

[5] Wojnarowski K Podobiński P Cholewińska P Smoliński J Dorobisz K. Impact of estrogens present in environment on health and welfare of animals. Animals. (2021) 11:2152. doi: 10.3390/ani11072152 34359280 PMC8300725

[6] Sharma M Kumar K Dubey KK. Disposal of unused antibiotics as household waste: a social driver of antimicrobial resistance. Environ Qual Manag. (2021) 30:127–40. doi: 10.1002/tqem.21744

[7] Attrah M Elmanadely A Akter D Rene ER. A review on medical waste management: treatment, recycling, and disposal options. Environments. (2022) 9:146. doi: 10.3390/environments9110146

[8] Chisholm JM Zamani R Negm AM Said N Abdel Daiem MM Dibaj M. Sustainable waste management of medical waste in African developing countries: a narrative review. Waste Manag Res. (2021) 39:1149–63. doi: 10.1177/0734242X211029175 34218734 PMC8488638

[9] Dioha MO Kumar AJR. Exploring sustainable energy transitions in sub-Saharan Africa residential sector: the case of Nigeria. Environ Sci Eng Econ. (2020) 117:109510. doi: 10.1016/j.rser.2019.109510

[10] Githinji MJ Njogu PM Nganga Z M K. Development and application of integrated indicators for assessing healthcare waste Management Systems in Kenyan Hospitals. Open J Appl Sci. (2024) 14:1080–120. doi: 10.4236/ojapps.2024.144072

[11] Iosue S. Comparative study of pharmaceutical waste disposal in Ethiopia, Kenya, Sudan and Uganda. (2020).

[12] Nepal S Giri A Bhandari R Chand S Nepal S Aryal S. Poor and unsatisfactory disposal of expired and unused pharmaceuticals: a global issue. Curr Drug Saf. (2020) 15:167–72. doi: 10.2174/1574886315666200626164001 32589562

[13] Salvia G Zimmermann N Willan C Hale J Gitau H Muindi K. The wicked problem of waste management: an attention-based analysis of stakeholder behaviours. J Cleaner Product. (2021) 326:129200. doi: 10.1016/j.jclepro.2021.129200 PMC8609182 34866810

[14] Angi’enda S Bukachi S. Household knowledge and perceptions on disposal practices of unused medicines in Kenya. J Anthropol Archaeol. (2016) 4:4. doi: 10.15640/jaa.v4n2a1

[15] Ayele Y MJ M. Assessment of knowledge, attitude and practice towards disposal of unused and expired pharmaceuticals among community in Harar city, eastern Ethiopia. J Pharm Policy Pract. (2018) 11:27. doi: 10.1186/s40545-018-0155-9 30459955 PMC6236888

[16] Jember E Feleke A Debie A G A. Self-medication practices and associated factors among households at Gondar town, Northwest Ethiopia: a cross-sectional study. BMC Res Notes. (2019) 12:1–7. doi: 10.1186/s13104-019-4195-2 30890186 PMC6425615

[17] Bogale Kassie A Mengie Ayele T Mekonnen Agidew M. Assessment of Dispensing Malpractice in Community Drug Retail Outlets in South Gondar Zone, Northwest Ethiopia: A Simulated Patient Experience. Integr Pharm Res Pract. (2023) 12:171–83. doi: 10.2147/IPRP.S416830 37522068 PMC10386832

[18] Kinrys G Gold AK Worthington JJ AA N. Medication disposal practices: increasing patient and clinician education on safe methods. J Int Med Res. (2018) 46:927–39. doi: 10.1177/0300060517738681 29322845 PMC5972255

[19] Haughey CW Lawson D Roberts K Santos M Spinosa S. Safe Medication Disposal. Home Healthc Now. (2019) 37:106–10. doi: 10.1097/NHH.0000000000000719 30829787

[20] Kahsay H Ahmedin M Kebede B Gebrezihar K Araya H Tesfay D. Assessment of knowledge, attitude, and disposal practice of unused and expired pharmaceuticals in community of Adigrat City, Northern Ethiopia. (2020). 10.1155/2020/6725423 PMC7178471 32351582

[21] Orina CN. Assessment of disposal practices of pharmaceutical waste among households within Nakuru town, Nakuru County. Kenya: Egerton University (2018).

[22] Kaloi GA Suheryani I Ghoto MA Mughal U-U-R Sultana R Tabassum R. Awareness towards Disposal of Unused Medication in District Shaheed Benazirabad Sindh. J Pharma Res Int. (2021) 33:41–9. doi: 10.9734/jpri/2021/v33i54B33763

[23] Rogowska J Zimmermann A. Household pharmaceutical waste disposal as a global problem—A Review. Int J Environ Res Public Health. (2022) 19:5798. doi: 10.3390/ijerph192315798 36497873 PMC9737308

[24] Hajj A Domiati S Haddad C Sacre H Akl M Tawil S. Assessment of knowledge, attitude, and practice regarding the disposal of expired and unused medications among the Lebanese population. J Pharm Policy Pract. (2022) 15, 15:107:107. doi: 10.1186/s40545-022-00506-z 36585685 PMC9802024

[25] Nairat LL Abahri NA Hamdan YA Abdel-Khaliq RT Odeh SM Abutaha S. Assessment of practices and awareness regarding the disposal of unwanted pharmaceutical products among community pharmacies: a cross-sectional study in Palestine. BMC Health Serv Res. (2023) 23:1035. doi: 10.1186/s12913-023-09888-5 37759203 PMC10537554

[26] Bekker CL Gardarsdottir H Egberts AC Bouvy ML Van den Bemt BJJP. Pharmacists’ activities to reduce medication waste: an international survey. Pharmacy. (2018) 6:94. doi: 10.3390/pharmacy6030094 30158484 PMC6165518

[27] Showande SJ Laniyan MW. Patient medication counselling in community pharmacy: evaluation of the quality and content. J Pharmaceuti Policy Practice. (2022) 15:103. doi: 10.1186/s40545-022-00502-3, PMID: 36527122 PMC9758914

[28] Abdul Kadir NF Suhaimi NS Balakrishnan M Wan Zaidi WN Fai SC. Assessment on knowledge, attitude and practice of patients in Hospital Tapah regarding disposal of unused and expired medicines. Malaysian J Pharmaceut Sci. (2021) 19:1–18. doi: 10.21315/mjps2021.19.1.1

[29] Zairina E Azzahrya AB Nugraheni G Sulistyarini AJPE. Knowledge, attitudes, and practices for using and disposing of antibiotics: a cross-sectional study at an Indonesian community. Pharm Educ. (2023) 23:110–5. doi: 10.46542/pe.2023.234.110115

[30] Nakiganda R Katende F Natukunda F Asio GJ Ojinga W Bakesiga A. (2023). Disposal of Unused Medicine among Health Professions Students at Makerere University: Knowledge, Practices and Barrier. *Res Sq* [Preprint]. doi: 10.21203/rs.3.rs-2525937/v1

[31] Dhande P Borulkar R Dombale Y. Unmasking the awareness and attitude regarding disposal of unused/expired medicines-a study from Metropolitan City, India. Bharati Vidyapeeth Med J. (2023) 3:18–25. doi: 10.56136/BVMJ/2023_01088

[32] Andrews JA Brown LJ Hawley MS A A. Older adults’ perspectives on using digital technology to maintain good mental health: interactive group study. J Med Internet Res. (2019) 21:e11694. doi: 10.2196/11694 30758292 PMC6391644

[33] Asmamaw G Agedew T Tesfaye B Sasamo S Gena S Argeta M. Prevalence of leftover medicines, disposal practices, and associated factors in Arba Minch town, Southern Ethiopia. SAGE Open Med. (2023) 11:205031212311582. doi: 10.1177/20503121231158214 PMC10021103 36935887

[34] Debrah JK Vidal DG Dinis MAPJR. Raising awareness on solid waste management through formal education for sustainability: a developing countries evidence review. Recycling. (2021) 6, 6:6:6. doi: 10.3390/recycling6010006

